# Polycaprolactone Impregnated 3D Printed Nanohydroxyapatite for Sinus Augmentation: A Randomized Controlled Trial

**DOI:** 10.1002/cre2.70237

**Published:** 2025-10-07

**Authors:** Poommarin Thammanoonkul, Suwit Limpattamapanee, Faungchat Thammarakcharoen, Jintamai Suwanprateeb, Hyun‐Chang Lim, Borvornwut Buranawat

**Affiliations:** ^1^ Center for Implant Dentistry and Periodontics Faculty of Dentistry and Research Unit in Innovations in Periodontics, Oral Surgery and Advanced Technology in Implant Dentistry Thammasat University Bangkok Thailand; ^2^ Nakhon Ratchasima Provincial Public Health Office Nakhon Ratchasima Thailand; ^3^ Biofunctional Materials and Devices Research Group National Metal and Materials Technology Center, National Science and Technology Development Thammasat University Bangkok Thailand; ^4^ Thammasat University Center of Excellence in Computational Mechanics and Medical Engineering Thammasat University Bangkok Thailand; ^5^ Department of Periodontology College of Dentistry, Periodontal‐Implant Clinical Research Institute, Kyung Hee University Medical Center Kyung Hee University Seoul Korea

**Keywords:** bone regeneration, maxillary sinus floor elevation, polycaprolactone impregnated 3D printed nano‐hydroxyapatite, randomized clinical trial

## Abstract

**Objective:**

To compare the effect of polycaprolactone impregnated 3D printed nano‐hydroxyapatite (3DPHA‐PCL) with bovine bone substitute material (BB) in lateral maxillary sinus floor elevation (MSFE).

**Materials and Methods:**

Lateral MSFE with two bone substitute materials was randomly performed in two centers: group BB (*n* = 11 sinuses) or group 3DPHA‐PCL (*n* = 11 sinuses). Lateral MSFE with two bone substitute materials was performed on 21 participants across two centers, resulting in a total of 22 sinuses analyzed. The sinuses were randomly allocated into two groups: group BB (11 sinuses) and group 3DPHA‐PCL (11 sinuses). Cone beam computed tomography (CBCT) was taken before (T0) and immediately after MSFE (T1), at 6 months (implant placement; T2), and 1 year (T3). Dimensional stability of the augmentation was analyzed using serial CBCT scans. At the time of implant placement, bone core biopsy was performed, followed by microcomputed tomographic (micro‐CT) and histomorphometric analyses.

**Results:**

Based on the superimposed CBCT images between T1 and T2, the augmented height and volume decreased in both groups without a statistically significant difference between the groups (−0.48 ± 1.01 mm, −53.9 ± 117.8 mm^3^ in group BB vs. −0.39 ± 0.44 mm, −40.8 ± 101.2 mm^3^ in group 3DPHA‐PCL, *p* > 0.05). The percentage of newly formed bone was statistically significantly lower in group 3DPHA‐PCL (15.7 ± 7.5% histomorphometrically, 16.7 ± 7.5% in micro‐CT) than group BB (25.6 ± 7.2%, 26.3 ± 4.1%) (*p* < 0.05 in both methods). Two implants failed in the 3DPHA‐PCL group, while no failures in the BB group.

**Conclusions:**

Dimensional stability of the augmented bone was comparable between the groups. However, group 3DPHA‐PCL demonstrated inferior new bone formation and implant survival compared to group BB. Long‐term follow‐up is warranted to monitor the behavior of 3DPHA‐PCL.

## 1 Introduction

The challenge in the posterior maxilla for implant treatment is predominantly maxillary sinus pneumatization (H. C. Lim et al. 2021). The pneumatization reduces available bone height for implants. According to available bone height (threshold value of 4−5 mm), either transcrestal or lateral maxillary sinus floor elevation (MSFE) is chosen for establishing a hard tissue envelope in the sinus for the implants (Misch 1987; Wallace and Froum 2003). The predictability of MSFEs was well demonstrated in numerous studies (W.‐B. Park et al. 2024; Pjetursson et al. 2008; Tan et al. 2008).

A variety of bone grafting materials can be used for MSFE (Al‐Nawas and Schiegnitz 2014; Danesh‐Sani et al. 2017; Shanbhag et al. 2014; Starch‐Jensen et al. 2018). Although autologous bone is considered the gold standard, it is rarely used for MSFE nowadays. This shift may be due to donor site morbidity, the fast resorption tendency (Jensen et al. 2006), and the high regenerative potential of the maxillary sinus itself. Instead, alternative materials, such as xenogeneic, allogenic, and alloplastic bone substitute materials, are more frequently used. Indeed, a recent systematic review and a Bayesian network meta‐analysis demonstrated no superiority of autologous bone to other substitute materials (Al‐Nawas and Schiegnitz 2014; Trimmel et al. 2021). Each substitute material possesses different characteristics in terms of resorption, bone‐forming mechanism, and physical properties. Within the context of MSFE, those substitute materials seem to be successful irrespective of their origins (Starch‐Jensen et al. 2018). However, xenogeneic and allogenic bone substitutes may be limited due to insufficient osteoinductivity, the possibility of disease transmission, degradation rate, and religious beliefs.

One of the research trends in regenerative dental treatment is to use three‐dimensional (3D) printing technology (Ivanovski et al. 2023). Previously, 3D printing technology was combined with low‐temperature phase transformation techniques, developing 3D printed hydroxyapatite bone substitutes (3DPH) (Suwanprateeb et al. 2010; Suwanprateeb et al. 2012). These substitutes exhibited several unique properties, such as nanostructure, low crystallinity, degradability, and high wicking ability, which contrasted with typical high‐temperature sintered HA. Preclinical and clinical investigations have demonstrated promising outcomes of those materials within the context of ridge preservation and lateral ridge deficiency (Kijartorn et al. 2017; Kijartorn et al. 2022; Mekcha et al. 2023). However, the mechanical strength of 3DPH was relatively low, potentially limiting its applicability to withstand load or stress in certain applications. Additionally, the material's brittleness occasionally made clinical handling challenging. Such limitations were particularly associated with high porosity due to calcium phosphate crystal precipitation during processing.

To address this drawback, a polycaprolactone (PCL) infiltrated 3DPH (3DPHA‐PCL) was developed (Suwanprateeb et al. 2016). PCL is one of the widely used synthetic polymers in the craniofacial region. It is known that PCL is highly biocompatible, biodegradable, and suitable for 3D printing. (Ivanovski et al. 2023). Furthermore, PCL can initially provide structural integrity to maintain the augmented space while also offering channels for tissue ingrowth through its porosity (Costa et al. 2014; Williams et al. 2005). Then, a degradation of PCL contributes to the porous architecture necessary for tissue ingrowth. 3DPHA‐PCL can be fabricated in various shapes. When produced in a spherical form (approximately 2 mm in diameter) using a modified proprietary technique, the material exhibits a highly porous structure with interconnected pores. This granule‐type 3DPHA‐PCL may be suitable for MSFE due to its applicability via a lateral access window and its structural stability.

The aim of the present study was to compare the effect of 3DPHA‐PCL with bovine bone substitute material (BB) in lateral MSFE. The null hypothesis was that, in the context of lateral MSFE, the newly developed 3DPHA‐PCL material is inferior to commercial BB in terms of new bone formation.

## 2 Materials and Methods

### 2.1 Study Design

The present study was designed as a multi‐center pilot single‐blinded randomized clinical trial in accordance with the Declaration of Helsinki and the International Conference on Harmonization (ICH) for Good Clinical Practice (GCP). The research protocol of this study was approved by the research ethical committee of Thammasat University (COA number 053/2564) and registered in the Thai Clinical Trials Registry (https://www.thaiclinicaltrials.org/show/TCTR20210622003). The CONSORT guidelines were observed.

### 2.2 Study Population

Patients needing lateral MSFE before implant placement in the posterior maxilla were recruited at the Implantology clinic, Faculty of Dentistry, Thammasat University, and Nakhonrachasima Health Provincial office. Before commencing the study, the patients were informed of the study's purpose, materials, and procedures. Informed consents were obtained from all patients. The following inclusion and exclusion criteria were applied.

#### 2.2.1 Inclusion Criteria

- Adult patients (age > 19 years old).
- Residual bone height between the ridge crest and the sinus floor ≤ 4 mm, assessed by cone‐beam computed tomography (CBCT) before lateral MSFE.
- Good general health (ASA class 1, 2).
- No history of allergy or hypersensitivity to the study materials.
- Signed informed consent.

#### 2.2.2 Exclusion Criteria

- Medical conditions and medications that would contraindicate oral surgery.
- History of malignancy, radiotherapy, and chemotherapy in the head and neck areas.
- Sinus pathologies contraindicate MSFE.
- Heavy smoker (≥ 10 cigarettes per day).
- Untreated and uncontrolled periodontal diseases.
- Pregnancy and lactation.
- Unwillingness to return for the follow‐up.

### 2.3 Study Group

- Group 3DPHA‐PCL: lateral MSFE using PCL impregnated 3D printed nanohydroxyapatite (3DPHA‐PCL, granule size: 2.0 mm, MTEC, Thailand).
- Group BB: lateral MSFE using BB (Straumann Xenograft, granule size: 1.0–2.0 mm, Straumann AG, Switzerland).

### 2.4 Fabrication and Characterization of 3DPHA‐PLC

**Figure 1:**
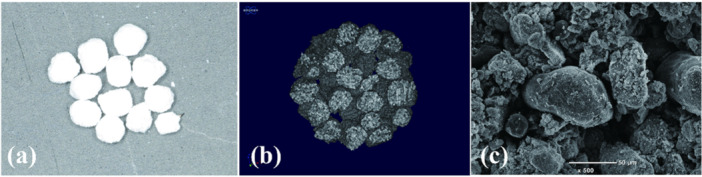
Characteristics of 3DPHA‐PCL. (a) Clinical view of 3DPHA‐PCL particles. (b, c) Scanning electron microscopic images of the particle.

Fabrication process of 3DPHA‐PCL was previously described elsewhere (Suwanprateeb et al. 2010; Thammarakcharoen et al. 2024). Briefly, spherical specimens (2 mm in diameter) were fabricated using a 3D printing machine with calcium sulfate‐based powder. These were soaked in a disodium hydrogen phosphate solution at 100°C for 48 h to transform into hydroxyapatite (3DPHA). The HA was then soaked in a PCL solution (50% PCL by weight) at 50°C for 15 min to create the 3DPHA‐PCL composite. The 3DPHA‐PCL granules exhibited a rough texture and featured a highly porous structure with interconnection by the connection of hydroxyapatite (HA) crystals that have been either coated or infiltrated with PCL. These granules possess a porosity of 60.67% and demonstrate a compressive load resistance of 7.55 ± 1.71 N. The samples were cleaned, dried, and sterilized by ethylene oxide gas (Figure 1). The details of the physicochemical properties of 3DPHA‐PCL and bovine bone (BB) are presented in the Supporting Information S1: Table S1.

### 2.5 Sample Size Calculation

Sample size was calculated by using G*Power software version 3.1. Based on the histomorphometrically new bone formation in the reference study (Pichotano et al. 2019), the effect size was deduced to 1.267. An *α* level and a study power were set to 0.05% and 80%, respectively. As a result, a minimum of 11 sites were required in each group. Considering the dropout rate (10%−15%), 12 sites were aimed to be included in the present trial.

### 2.6 Randomization and Allocation Concealment

Twenty‐four eligible patients were allocated to either the test or control group using a computer‐generated randomization sequence (block size = 4). The allocation sequence was concealed in sealed, opaque envelopes, which were opened in numerical order by another investigator who was not involved in this study. The patients were not informed of which group they were assigned to. However, due to the visible differences in the materials, the surgeons could not be blinded to the assignment.

### 2.7 Clinical Procedures

#### 2.7.1 Lateral MSFE

**Figure 2:**
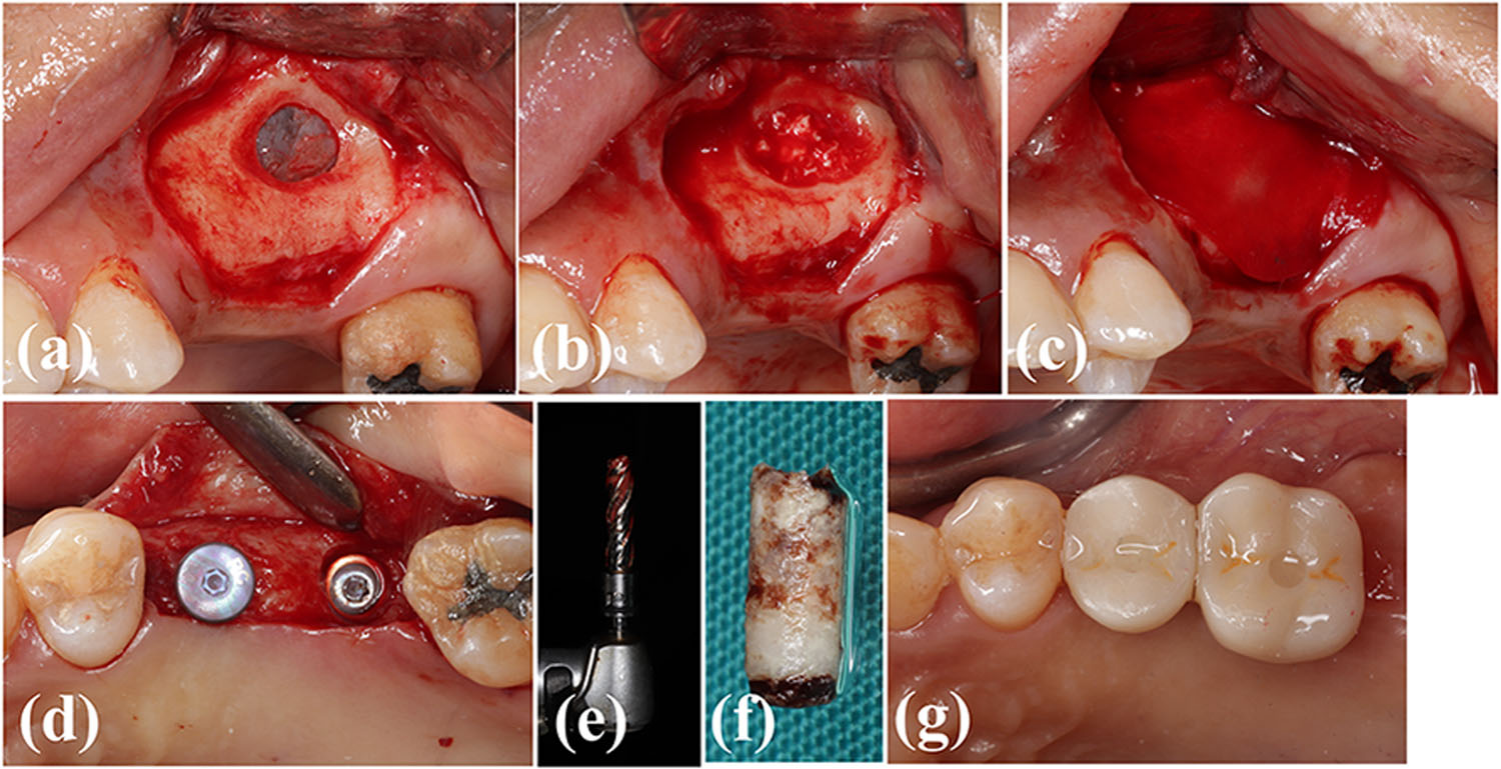
Clinical photographs of the surgery. (a) A bony access window was created on the lateral wall of the maxillary sinus. (b) 3DPHA‐PCL was filled in the sinus after detaching the sinus membrane. (c) A collagen membrane was applied to cover the access window. (d) Dental implants were placed on #15 and #16 areas. (e) A bone core biopsy was performed during drilling. (f) The obtained specimen. (g) An implant prosthesis was delivered (#17 was extracted).

Before the lateral MSFE, the patients were asked to rinse their mouths with chlorhexidine solution (0.12% for 1 min). Under local anesthesia with 4% articaine (containing 1:100,000 epinephrine), a mucoperiosteal trapezoidal flap was raised to expose the lateral wall of the maxillary sinus. A bony access window was made with a diamond‐coated dome‐shaped drill (DASK kit, Dentium, Seoul, Korea), followed by gentle detachment of the sinus membrane from the sinus bone walls. After detaching the membrane, an independent investigator opened the sealed envelope. Then, according to the group assignment, either 3DPHA‐PCL or BB was packed gently. The bony window was covered by a collagen membrane (Lyoplant, Aesculap AG, Germany Bioland). The flap was closed using a nylon suture material (ETHILON 5‐0, Ethicon, New Jersey, US) (Figure 2). Postsurgical (T1) radiographic examination was performed.

Postsurgical medication included amoxicillin‐clavulanate (GSK, London, UK), Ibuprofen (OSI, Bangkok, Thailand), and Paracetamol (TNP, Nonthaburi, Thailand) for 5 days. The patients were recommended to use chlorhexidine mouthwash until suture removal. The sutures were removed 2 weeks post‐surgery.

#### 2.7.2 Implant Placement

After 6 months (T2), implant placement was performed. Before implant drilling, a bone core biopsy was obtained from an implant site using a trephine bur (inner diameter: 2.9 mm, Meisinger, Neuss, Germany). The trephine bur was inserted to a depth of approximately 10 mm from the bone crest. The obtained specimens were placed in a fixative (10% neutral formalin). Implant drilling was then performed according to the manufacturer's guidelines. Subsequently, bone‐level implants were placed, and healing abutments were connected (Figure 2). Implant stability was checked using a modified damping capacity analysis device (AnyCheck IMT‐100, Neobiotech, Seoul, Korea). The same postoperative regimen (as the prior surgery) was performed.

#### 2.7.3 Prosthetic Phase

After another 3 months, implant stability was checked again to commence the prosthetic phase. Impression was taken using polyether impression material (3M Impregum, Neuss, Germany), and a screw‐retained zirconia implant prosthesis was inserted 2 weeks later (Figure 2).

#### 2.7.4 Follow‐Up

At 3 months post‐final prosthesis insertion (T3; at 1 year post‐lateral MSFE), the patients were re‐called to check the implants and collected radiographic and clinical data (Figure 2). Representative CBCT images for BB and 3DPHA‐PCL groups at T0, T1, T2, and T3 are shown in Figure S1.

### 2.8 Outcome Measures

#### 2.8.1 Primary Outcome

- Percentage of newly formed bone, assessed by histomorphometry.

#### 2.8.2 Secondary Outcomes

- Percentage of residual material in the biopsy specimens, assessed by histomorphometry.
- Percentage of newly formed bone and residual material in the biopsy specimens, assessed by microcomputed tomography (micro‐CT).
- Vertical bone height at each time point (T0, T1, T2, T3) and changes between the time points, assessed using CBCT.
- Augmented bone volume at each time point (T0, T1, T2, T3) and changes between the time points, based on CBCT.
- Implant stability (IST), using a modified damping capacity analysis device.
- Peri‐implant clinical parameters, such as sulcus bleeding index (SBI), plaque index (PI), and probing pocket depth (PD).
- Marginal bone level: the distance from the implant shoulder to the first bone‐to‐implant contact on the periapical radiographs by the parallel‐technique, measured using ImageJ software (NIH, Bethesda, MD, USA).

### 2.9 Analyses

Two modes of analysis (micro‐CT and histomorphometry) for tissue contents in the bone core specimens were performed. The decalcification procedure to produce the histological slides can partially dissolve calcium contents from the specimens and form voids in the histological sections, which may cause misinterpretation, especially for 3DPHA‐PCL. Thus, through those two modes, a more accurate assessment of the behaviors of the materials is feasible. Regarding the stability of the materials, CBCT data were linearly and volumetrically evaluated.

The examiner responsible for performing the histomorphometric and radiographic analyses was blinded to the group assignments throughout the evaluation process, thereby reducing the risk of assessment bias and enhancing the reliability of the study outcomes.

#### 2.9.1 Micro‐CT Analysis

**Figure 3:**
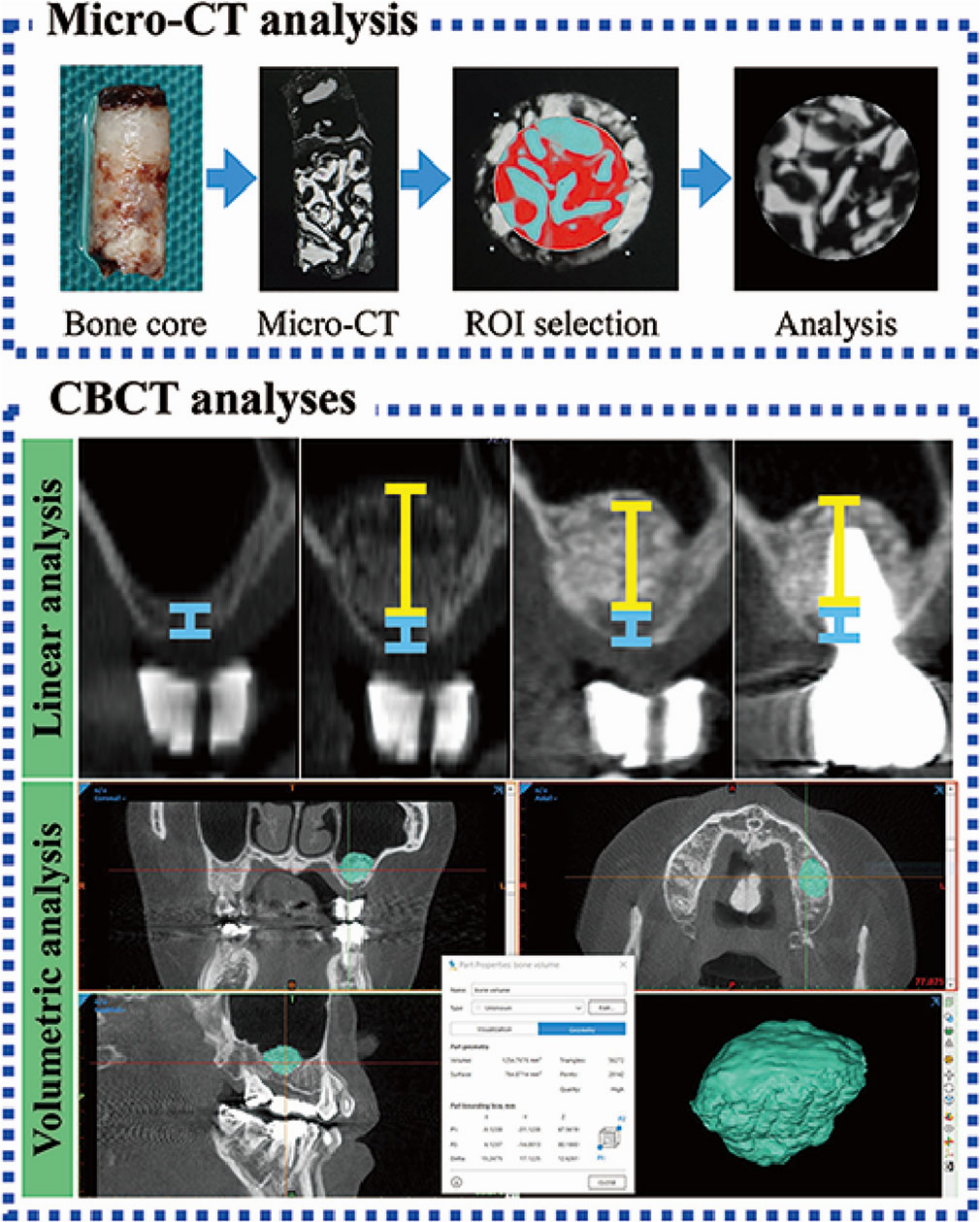
Microcomputed tomographic and conebeam computed tomographic analyses. Blue and yellow lines indicate residual native bone and augmented bone heights, respectively.

Before histological processing, all bone core specimens were scanned by micro‐CT (Skyscan 1275, Bruker microCT, Kontich, Belgium) to quantify 3D microarchitecture. The shooting condition was set at the following: 50 kV, 80 μA, a 0.5 mm aluminum filter, and a 360° rotation with three‐frame averaging. The raw images were reconstructed using NRecon software (Bruker microCT, Kontich, Belgium). For the reconstruction parameters, a ring artifact correction was set at 3, and the beam hardening correction was set at 61%. Following reconstruction, a circular region of interest (ROI) with a diameter of 2.5 mm was established from the center point. Subsequently, a cylindrical shape of the volume of interest (VOI) was set between 1 and 4 mm from the sinus floor (3 mm in height) (Figure 3). To discern the specific tissue components, specific grayscales were applied for 3DPHA‐PCL (56‐100), BB (161‐255), and newly formed bone (101−160). Then, the percentage of newly formed bone and residual material within each specimen was calculated.

#### 2.9.2 Histomorphometry

After micro‐CT scanning, the specimens were decalcified in 10% EDTA (pH 7.4) over 7 days, followed by dehydration in a series of ethanol solutions and embedment in paraffin blocks. Serial sections (3−5‐μm‐thick) were made by cutting along the center of the specimen and parallel to the long axis (Leica RM2235, Leica Biosystems Nussloch GmbH, Germany). The central‐most section of each sample was stained with hematoxylin and eosin (H&E). The obtained histological slides were examined under a light microscope equipped with a camera system (Nikon DS‐U3, USA) and processed into digital images. Histomorphometric analysis was then performed in the augmented area (excluding the native bone part) using an image analysis software (ImageJ software, NIH, Bethesda, MD, USA). The percentages of newly formed bone and residual bone substitute material were calculated.

#### 2.9.3 Radiographic Analyses

To analyze the same section at four time points, a radiographic guide stent was made for each site. Vertical bone heights between the ridge crest and the sinus floor (at T0) and between the ridge crest and the top of augmented bone (at T1, T2, and T3) were measured in the coronal sections of CBCT scans (Figure 3). The changes in VHs were calculated between time points. Moreover, the changes were calculated in percentages.

The obtained bone volume was measured using a computer software (MIMICS 25.0 software, Materialise Europe, World Headquarters, Leuven, Belgium) (Figure 3) (Zhang et al. 2022). Superimposition of 2 DICOM files (postoperative lateral maxillary sinus augmentation; T1−T3 and preoperative file; T0). Then, the grafting area was selected and calculated into mm^3^. The bone volume changes were calculated with respect to the initially augmented volume at T1.

### 2.10 Calibration

All measurements were performed by a single examiner (P.T.). The group assignment to the samples was not informed. Before the main analyses, the examiner was trained using selected samples that were not related to the present study. The training was supervised by a senior investigator (B.B.). After training sessions, the interclass correlation coefficient between two investigators was between 0.895 and 0.936 (*p* < 0.05).

### 2.11 Statistical Analysis

The data were analyzed using a statistical analysis software (GraphPad Prism 9.1.1, California, USA). The data were expressed as mean ± standard deviation. Normal distribution was tested by the Shapiro−Wilk test. Then, independent *t*‐tests were used for statistically significant differences between the two groups. A *p* < 0.05 was considered statistically significant. Additionally, 95% confidence intervals (CI) and Cohen's *d* effect size for between‐group differences were calculated.

## 3 Results

**Figure 4:**
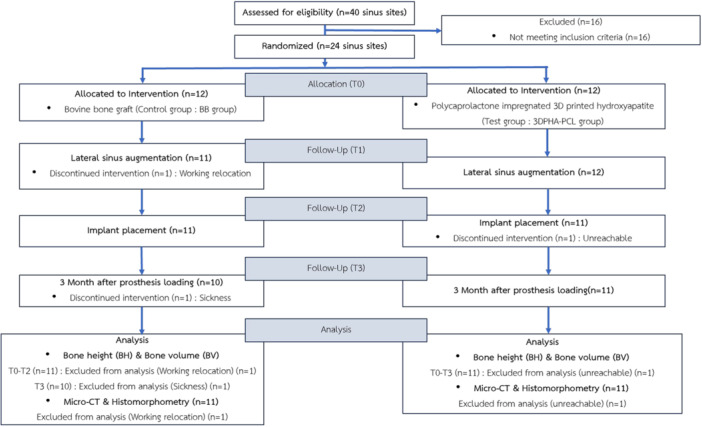
Consort flowchart.

Initially, 40 patients were screened, confirming the eligibility of 22 patients with 24 sinuses. One patient in group BB and one in group 3DPHA‐PCL required bilateral MSFE. During the study, three patients were lost due to moving and loss of contact. Twenty patients underwent implant placement, leading to 22 sinuses in the analyses (17 sinuses in 16 patients at Thammasat University, five sinuses in four patients at Nakhon Ratchasima Health Provincial office) (Figure 4). The study period was from December 2022 to October 2024.

### 3.1 Demographic Information

Table 1 presents the demographic information of the included patients. Mean ages of the patients were 60.7 ± 10.1 years old in group BB (three males and eight females) and 52.36 ± 14.22 in group 3DPHA‐PCL (six males and five females). ANCOVA adjusting for age and gender confirmed that these variables did not significantly affect the primary outcome (*p* > 0.15). Most of the implant sites were molar areas (*n* = 20). Three implant systems were used to replace missing teeth (Straumann BLT [Basel, Switzerland]: 14, Neobiotech [Seoul, Korea]: 3, Bego [Bremen, Germany]: 5). Implant diameters ranged from 3.5 to 5.5 mm, and lengths varied from 8 to 11.5 mm. Sinus membrane perforation occurred at two sites, which were managed using a collagen membrane. After lateral MSFE, all sites were healed without adverse events, such as sino‐nasal infection, persistent pain, and wound dehiscence. All implants presented adequate stability (> 80% of the IST value) for prosthetic loading.

**Table 1:** Patients' demographic data and clinical characteristics.

	Group BB (*n* = 11)	Group 3DPHA‐PCL (*n* = 11)	Total (22)
Age (mean ± SD)	60.7 ± 10.1	52.4 ± 14.2	56.6 ± 12.8
Gender (%)			
Male	3 (27.3)	6 (54.6)	9 (40.9)
Female	8 (72.7)	5 (45.5)	13 (59.1)
Tooth type (%)			
Premolar	—	2 (18.2)	2 (9.1)
Molar	11 (100)	9 (81.8)	20 (90.9)
Implant (%)			
Brand			
Straumann	8 (72.7)	6 (54.6)	14 (63.6)
Neobiotech	—	3 (27.3)	3 (13.6)
Bego	3 (27.3)	2 (18.2)	5 (22.7)
Diameter			
3.5	—	1 (9.1)	1 (4.5)
4.0	—	1 (9.1)	1 (4.5)
4.1	1 (9.1)	1 (9.1)	2 (9.1)
4.5	2 (18.2)	—	2 (9.1)
4.8	7 (63.6)	5 (45.5)	12 (54.6)
5.0	—	2 (18.2)	2 (9.1)
5.5	1 (9.1)	1 (9.1)	2 (9.1)
Length			
8.0	1 (9.1)	—	1 (4.8)
8.5	—	1 (9.1)	1 (4.8)
10.0	8 (72.7)	10 (90.9)	18 (85.7)
11.5	2 (18.2)	—	1 (4.8)
Membrane perforation	1 (9.1)	1 (9.1)	2 (9.1)
Implant survival (%)	10/10 (100)	9/11 (81.8)	19 (90.5)

*Note:* Note that one patient in group BB was lost at T3 due to sickness.

Abbreviations: Group BB, lateral MSFE using bovine bone substitute material; Group 3DPHA‐PCL, lateral MSFE using polycaprolactone impregnated 3D printed nanohydroxyapatite.

### 3.2 Micro‐CT Outcomes

**Figure 5:**
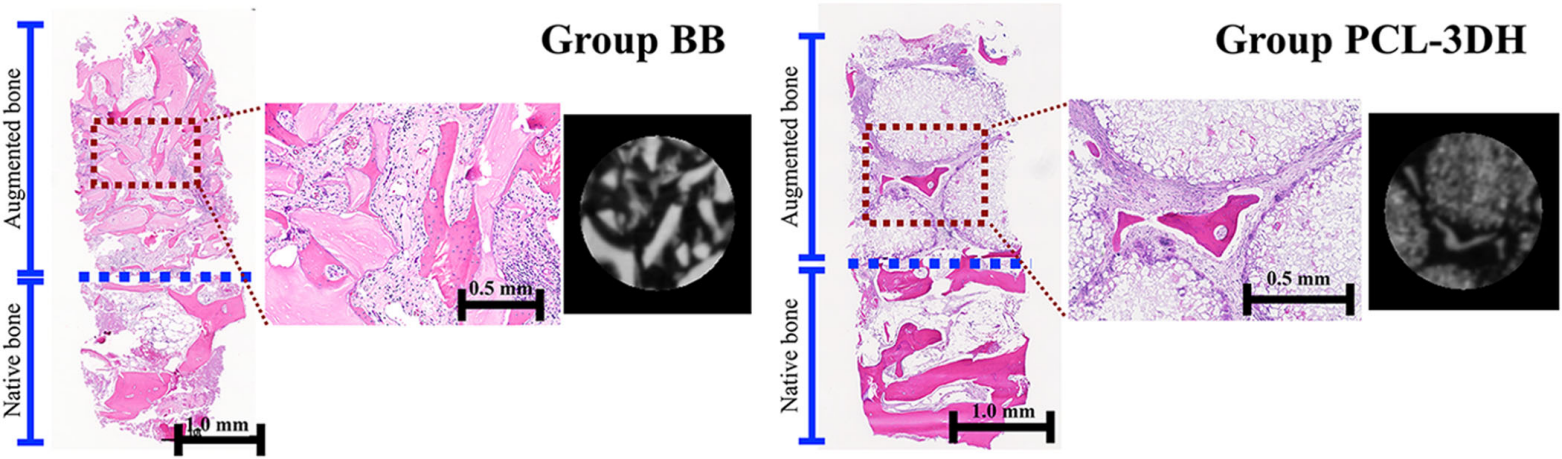
Representative histological and micro‐CT images of groups BB and 3DPHA‐PCL. Group BB, lateral MSFE using bovine bone substitute material; Group 3DPHA‐PCL, lateral MSFE using polycaprolactone impregnated 3D printed nanohydroxyapatite.

Micro‐CT reconstruction revealed the 3D structure of mineralized bone. In group BB, the trabecular bone was in contact with bone substitute particles, whereas there were gaps between the trabecular bone and the bone substitute (Figure 5). The amount of newly formed bone was statistically significantly greater in group BB than in group 3DPHA‐PCL (26.3 ± 4.1% vs. 16.7 ± 7.5%, *p* < 0.05) (Table 2).

**Table 2:** Histomorphometric and microcomputed tomographic analyses.

	Micro CT		Histomorphometry	
	NB	RM	NB	RM
Group BB (*n* = 11)	26.3 ± 4.06	32.3 ± 13.5	25.6 ± 7.2	30.9 ± 9.1
Group 3DPHA‐PCL (*n* = 11)	16.7 ± 7.5	30.1 ± 12.2	15.7 ± 7.5	33.2 ± 15.2
*p* value	0.0014*	0.6934	0.0049*	0.6794
95% CI	−14.93 to −4.202	−13.65 to 9.26	−16.44 to −3.362	−8.883 to 13.35
Effect size (*d*)	1.59	0.17	1.35	−0.18

*Note:* The effect sizes are calculated as (group BB−group 3DPHA‐PCL)/pooled SD, with interpretation based on magnitude (negligible: [*d*] < 0.2; small: 0.2 ≤ [*d*] < 0.5; medium: 0.5 ≤ [*d*]< 0.8; large: [*d*]≥ 0.8).

Abbreviations: Group BB, lateral MSFE using bovine bone substitute material; Group 3DPHA‐PCL, lateral MSFE using polycaprolactone impregnated 3D printed nanohydroxyapatite; NB, newly formed bone; RM, residual bone substitute material.

*p Values (statistically significant at the level of of *p* < 0.05) for differences in percentage of a new bone formation and residual bone graft between two groups.

### 3.3 Histological and Histomorphometric Outcomes

The specimens consisted of native bone parts and a composite of newly formed bone and bone substitute material (augmented part). In group BB, non‐lamellated newly formed bone with osteocytes and an osteoid layer was observed predominantly on the bone substitute particles. In some parts of the specimens, the newly formed bone bridged adjacent particles. A few osteoclasts were observed on the particles. In 3DPHA‐PCL, newly formed bone was mainly found in the inter‐particular spaces. Osteoclast‐like multinucleated giant cells were found on the particles (Figure 5).

The percentage of newly formed bone was 25.6 ± 7.2% in group BB and 15.7 ± 7.5% in group 3DPHA‐PCL, which reached a statistically significant difference between the groups (*p* < 0.05). The percentage of residual bone substitute material was not statistically significantly different between the two groups (30.9 ± 9.1% vs. 33.2 ± 15.2%, *p* > 0.05) (Table 2).

### 3.4 Implant Stability

IST values at the time of implant placement showed no statistically significant difference between group BB and group 3DPHA‐PCL (69.4 ± 5.2 vs. 66.9 ± 4.0, *p* > 0.05). An increase in those values was observed at 3 months post‐implant placement. At that time, the difference between the groups became significant despite a small difference (85.8 ± 2.0 vs. 82.8 ± 2.7, *p* < 0.05).

### 3.5 Radiographical Outcomes

#### 3.5.1 Vertical Bone Height

The initial bone height (at T0) was 3.4 ± 1.2 mm in group BB and 3.4 ± 1.0 mm in group 3DPHA‐PCL (*p* > 0.05). At immediate lateral MSFE (T1), the height increased to 12.6 ± 2.3 mm in group BB and 13.1 ± 1.8 mm in group 3DPHA‐PCL (*p* > 0.05). Over time, the height in both groups decreased, and at 1 year (T3), the values were 12.1 ± 1.9 mm and 12.7 ± 1.6 mm in groups BB and 3DPHA‐PCL, respectively (*p* > 0.05) (Table 3).

**Table 3:** Vertical bone height and bone volume in the augmented sinuses.

	Group BB (*n* = 11 at T0, T1 & T2, *n* = 10 at T3)	Group 3DPHA‐PCL (*n* = 11 at T0, T1 & T2, *n* = 9 at T3)	*p* value	95% CI	Effect size (*d*)
Vertical bone height (mm)					
T0	3.4 ± 1.2	3.4 ± 0.9	0.9074	−0.99 to 0.88	0.00
T1	12.6 ± 2.3	13.1 ± 1.8	0.5384	−1.28 to 2.38	−0.24
T2	12.2 ± 2.0	13.0 ± 1.6	0.3473	−0.86 to 2.34	−0.44
T3	12.1 ± 1.9	12.7 ± 1.6	0.3877	−0.93 to 2.29	−0.34
Vertical bone height change (mm)					
T1‐T0	9.2 ± 2.8	9.77 ± 2.07	0.5754	−1.60 to 2.81	−0.23
T3‐T0	8.7 ± 2.5	9.4 ± 2.0	0.4753	−1.34 to 2.77	−0.31
T3‐T1	−0.48 ± 1.01	−0.39 ± 0.44	0.7171	−0.61 to 0.79	−0.11
Augmented bone volume (mm^3^)					
T1	1037.0 ± 232.5	1087.0 ± 273.7	0.6475	−175.6 to 276.1	−0.20
T2	1040.0 ± 224.6	1085.0 ± 244.3	0.6581	−163.8 to 253.7	−0.19
T3	965.1 ± 257	1046.0 ± 260.5	0.481	−155.4 to 318.0	−0.31
Augmented bone volume change (mm^3^)					
T2‐T1	2.8 ± 90.4	−2.5 ± 138.9	0.9164	−109.5 to 98.90	0.05
T3‐T2	−60.1 ± 37.9	−38.3 ± 74.7	0.4175	−33.24 to 76.82	−0.37
T3‐T1	−53.9 ± 117.8	−40.8 ± 101.2	0.7871	−86.93 to 113.1	−0.12

*Note:* The effect sizes are calculated as (Group BB−Group 3DPHA‐PCL)/pooled SD, with interpretation based on magnitude (negligible: [*d*] < 0.2; small: 0.2 ≤ [*d*] < 0.5; medium: 0.5 ≤ [*d*] < 0.8; large: [*d*] ≥ 0.8).

Abbreviations: Group BB, lateral MSFE using bovine bone substitute material; Group 3DPHA‐PCL, lateral MSFE using polycaprolactone impregnated 3D printed nanohydroxyapatite; T0, before lateral maxillary sinus floor elevation (MSFE); T1, immediately after lateral MSFE; T2, 6 months after lateral MSFE; T3, 1 year after lateral MSFE.

The gain of vertical bone height (the difference between T0 and T3) and the vertical changes in the augmented bone (the difference between T1 and T3) were not statistically significantly different (*p* > 0.05) (Table 3).

#### 3.5.2 Augmented Bone Volume

There were no statistically significant differences in the augmented bone volume and volume changes between the two groups (*p* > 0.05) (Table 3).

### 3.6 Implant‐Related Outcomes

#### 3.6.1 Implant Survival

One patient in group BB did not attend the T3 visit due to illness, resulting in 10 implants left. All implants in this group survived. In the 3DPHA‐PCL group, two implants failed between 1 and 2 months of loading. The suspected reasons for these failures were as follows: one patient had severe bruxism but refused to wear a nightguard, and in the other patient, the implant engagement in the native bone was minimal (residual bone height was 1.4 mm at T0, and the implant was placed 1 mm below the bone crest in the augmented site). Key findings were summarized in Supporting Information S1: Table 2.

#### 3.6.2 Peri‐Implant Clinical and Radiographic Parameters

Sulcus bleeding index, plaque index, probing depth, and marginal bone level did not show statistically significant difference between the groups (Table 4).

**Table 4:** Peri‐implant clinical and radiographic parameters at 3 months post‐loading.

	Group BB (*n* = 10)	Group 3DPHA‐PCL (*n* = 9)	*p* value	95% CI	Effect size (*d*)
SBI	0.50 ± 0.53 (10)	0.56 ± 0.53 (9)	0.8213	−0.46 to 0.57	−0.11
PI	0.80 ± 0.63 (10)	0.44 ± 0.53 (9)	0.2036	−0.92 to 0.21	0.62
PPD	3.10 ± 0.74 (10)	3.20 ± 0.63 (9)	0.7486	−0.56 to 0.81	−0.14
MB	0.17 ± 0.23 (10)	0.27 ± 0.18 (9)	0.3304	−0.11 to 0.30	−0.48

*Note:* The effect sizes are calculated as (Group BB−Group 3DPHA‐PCL)/pooled SD, with interpretation based on magnitude (negligible: [*d*] < 0.2; small: 0.2 ≤ [*d*]< 0.5; medium: 0.5 ≤ [*d*]< 0.8; large: [*d*] ≥ 0.8).

Abbreviations: Group BB, lateral MSFE using bovine bone substitute material; Group 3DPHA‐PCL, lateral MSFE using polycaprolactone impregnated 3D printed nanohydroxyapatite; MB, marginal bone level; PI, plaque index (PI); PPD, probing pocket depth; SBI, sulcus bleeding index.

## 4 Discussion

In the present study, the effectiveness of a novel biomaterial (PCL impregnated 3D printed nanohydroxyapatite; group 3DPHA‐PCL) was compared with a commercially available BB (group BB). Two materials presented similar stability of the augmentation during the study period. However, group 3DPHA‐PCL demonstrated inferior new bone formation (assessed by histomorphometry and micro‐CT) and lower implant stability compared to group BB.

A variety of bone substitute materials are used in implant treatment, including MSFE. Even though the existing materials demonstrated favorable outcomes, next‐generation materials are needed to further enhance bone healing and carry out multiple purposes, such as delivering antibiotics or growth factors. In the current study, we used PCL impregnated 3D printed nanohydroxyapatite. The other versions of this material were tested previously (Suwanprateeb et al. 2016), demonstrating promising clinical, radiographical, and histological outcomes.

PCL impregnated 3D printed nanohydroxyapatite in the present was designed in the spherical form with a diameter of 2 mm. Such a diameter was larger than that of conventionally used bone substitutes. Specifically, in the context of lateral MSFE, the particle size has been an issue. Despite heterogeneity, it seems that a higher number of studies supported using large particles (1.0–2.0 mm) compared to small particles (0.25–1.0 mm) on the basis of histomorphometric, radiographic, and immunohistochemical results. The bone substitutes with large particle size favored an increase in bone formation‐related markers and subsequent new bone formation. The percentage of newly formed material from the control material (particle size: 1.0–2.0 mm) in the present study was 25.6 ± 7.2% histomorphometrically and 26.3 ± 4.1% in micro‐CT, which is in line with other studies with the same particle size (Kamolratanakul et al. 2022; Krennmair et al. 2024; Testori et al. 2013).

However, the 3DPHA‐PCL in this study produced a smaller percentage of newly formed bone in both histomorphometric and micro‐CT analyses. This inferior new bone formation might be attributed to the incorporation of PCL into the nanohydroxyapatite material. Ideally, the degradation of biomaterial should be coupled with new bone formation, but the biodegradation of PCL seemed to take longer (Sun et al. 2006) than is suitable for endosinus bone formation. Moreover, the hydrophobicity of PCL might be one of the reasons for less new bone formation. Such a feature might have impaired cell adhesion and proliferation. In comparison with other studies, a lower percentage of newly formed bone was also noted in group 3DPHA‐PCL. For example, the results of studies using various synthetic bone substitute materials generally demonstrated more than 20% of newly formed bone (Belouka and Strietzel 2016; Bouwman et al. 2017; Cordaro et al. 2008; Ohayon 2014). The lower rate of new bone formation potentially carries the risk of lower bone‐to‐implant contact, which may affect the long‐term survival of the implants. However, this concern remains inconclusive due to the limited follow‐up period. Although the approximately 10% difference in new bone formation observed with 3DPHA‐PCL compared to BB indicates enhanced bone regeneration, it may not directly translate into implant survival or long‐term functional outcomes. Implant success is influenced by a complex interplay of factors beyond bone volume, including bone quality, surgical technique, implant design, patient health, and loading conditions. While greater new bone can contribute to better initial stability and osseointegration, implant survival rates do not solely depend on the percentage of new bone formed. Therefore, the observed difference should be viewed as a positive biological indicator rather than a definitive predictor of clinical success. Long‐term clinical studies evaluating implant survival and function are necessary to confirm any meaningful impact of this bone formation difference on patient outcomes.

In MSFE, the change of augmented dimension should be taken into account. In all studies, varying degrees of dimensional change were reported, and the change was predominantly a decreasing tendency. Immediately after MSFE, the augmented area contains a blood‐filled space, which can be present in the inter‐particular space and is a little separated from the mass of substitute material. Over time, this space seems to shrink with biologic resorption and pressure of the overlying sinus membrane. Moreover, bone substitute materials undergo degradation with new bone formation. Depending on such behavior, the augmented dimension can be affected. Interestingly, it was demonstrated that excessive grafting was related to more shrinkage (Pesce et al. 2021). Some studies reported a relatively high shrinkage of the augmented volume (18%−28%), (Kirmeier et al. 2008; Zhang et al. 2022) but others not; approximately 10%, (Krennmair et al. 2024) 5.8% and 6.6%, (Mordenfeld et al. 2016), and less than 1 mm (Cha et al. 2019; J. H. Park et al. 2023). In the present study, the dimensional changes of two bone substitute materials were in line with the latter studies, revealing high stability: less than 1 mm linearly and less than 4% volumetrically. However, dimensional stability should be further monitored.

Regarding methodology to evaluate graft stability, we used two types of analyses: linear and volumetric analyses. Conventionally, the longitudinal axis of the implant has been a reference for linear analysis, and with the use of such a reference, the changes of augmented bone in a selective area can be revealed. However, linear analysis has some limitations, as follows: (1) the changes in other parts relatively irrelevant to the implant may be difficult to quantify, (2) when the reference is absent, linear analysis may become inaccurate and hard to interpret. Thus, volumetric analysis can supplement the behavior of the graft in more detail. In the present study, the results from both analyses coincided in terms of small dimensional change, ensuring the high stability of the augmented dimension.

In the present study, implant stability was tested with a modified damping capacity analysis device. Several studies demonstrated a correlation between the current device (Anycheck) and other damping analysis devices (e.g., Periotest M) or the implant stability quotient by resonance frequency analysis (Kim et al. 2024; Lee et al. 2023; H. K. Lim et al. 2022). Damping analysis may not be as precise as resonance frequency analysis for evaluating the degree of osseointegration or borderline stability for enduring functional loading (Hürzeler et al. 1995), but it was suggested that the range between −4 and +2 or between −4 and −2 is needed for discerning clinically osseointegrated implants (Morris et al. 2003; Teerlinck et al. 1991). In one study, it was shown that the values of Periotest M between −4.25 and −5.29 corresponded to the values between 75.82 and 77.48 with Anycheck (H. K. Lim et al. 2022). Thus, it could be regarded that the implant stability values in both groups were clinically sufficient for inserting the implant prosthesis (85.8 ± 2.0 in group BB, 82.8 ± 2.7 in group 3DPHA‐PCL) despite a statistically significant difference between the groups. However, it should be considered that the performance of damping analysis devices is affected by striking direction, the height of the abutment, and peri‐implant bone condition more than resonance frequency analysis (Kang et al. 2023; O'Brien et al. 2024). Such a limitation might be related to implant failure in the group 3DPHA‐PCL. Although the suspected reasons for the failure were plausible (bruxism, thin native bone height) and may also be related to the 3DPHA‐PCL itself, especially its slower rate of bone formation, the values from the damping analysis device might not accurately capture the actual bone‐to‐implant engagement.

This study has several limitations. First, the follow‐up period was limited to 1 year. While such a timeframe provides valuable insights into the early behaviors of the applied materials, a more extended observation period is still needed to verify the long‐term effects of the materials (in terms of degradation and new bone formation). If the amount of newly formed bone does not increase, such a condition may not be suitable for bearing the occlusal load in the long term. Second, there were imbalances of patient distribution in terms of gender and centers. The uneven distribution might affect treatment outcomes. However, gender imbalance was hard to control, considering the limited time frame to complete the study. One may point out the center effect because one center treated four times more patients than the other. Such was due to the size of the center and the number of outpatients. However, considering the nature of the current intervention and the surgeons' experience, the difference in treatment results might not lead to a high disparity. Third, the implant brand was not homogeneous across patients. Different implant systems vary in surface properties, design, and connection types, which can influence clinical outcomes. Fourth, the statistical unit in the present study was the site. Even though bilateral sinuses were included in only two patients, this might lead to overestimation of the effective sample size. Fifth, the granule size differed between the groups. Although this variable may have influenced the treatment outcomes, the difference was inevitable due to the increased brittleness of 3DPHA‐PCL material in smaller particles.

In conclusion, 3DPHA‐PCL demonstrated clinical safety with favorable stability of the augmented dimension and implant. However, it was shown that new bone formation was retarded in the sinuses using 3DPHA‐PCL compared to those with commercial BB. Given that this pilot study involved a limited sample size and a follow‐up duration of only 1 year, the evidence generated is underpowered and should be interpreted with caution. To robustly confirm and extend these findings, larger‐scale studies with extended observation periods are necessary.

## Author Contributions

**Poommarin Thammanoonkul:** conceptualization, methodology, investigation, formal analysis, writing original draft. **Suwit Limpattamapanee:** clinical investigation, data curation. **Faungchat Thammarakcharoen:** biomaterial development, methodology, resources, writing – review and editing. **Jintamai Suwanprateeb:** biomaterial development, resources, writing – review and editing. **Hyun‐Chang Lim:** conceptualization, editing, supervision. **Borvornwut Buranawat:** study design, clinical supervision, funding acquisition, writing – review and editing.

## Conflicts of Interest

The authors declare no conflicts of interest.

## Supporting information


Supplemaentary Figure S1:

**Supplementary Table S1:** Physicochemical Properties of 3DPHA‐PCL vs Bovine Bone. **Supplementary Table S2:** Summary comparison of 3DPHA‐PCL *vs*. Bovine Bone in Maxillary Sinus Floor Elevation.

## Data Availability

The data that support the findings of this study are available on request from the corresponding author. The data are not publicly available due to privacy or ethical restrictions.
