# Supplementary figures and images for: Polycaprolactone Impregnated 3D Printed Nanohydroxyapatite for Sinus Augmentation: A Randomized Controlled Trial
Source: Clin Exp Dent Res. 2025 Oct 7;11(5):e70237. doi: 10.1002/cre2.70237 (PMC12502625; doi:10.1002/cre2.70237)

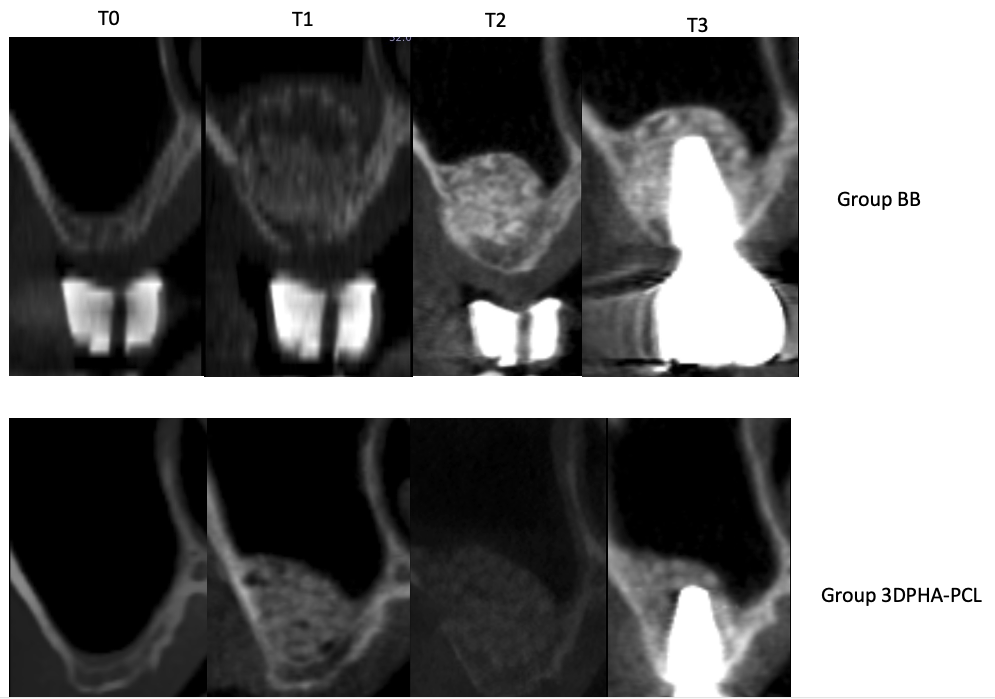

Supplement: Supplementary file 1 — Supplemaentary Figure S1: [file CRE2-11-e70237-s001.png]
