# Supplementary material for: Polycaprolactone Impregnated 3D Printed Nanohydroxyapatite for Sinus Augmentation: A Randomized Controlled Trial
Source: Clin Exp Dent Res. 2025 Oct 7;11(5):e70237. doi: 10.1002/cre2.70237 (PMC12502625; doi:10.1002/cre2.70237)
Supplement: Supplementary file 2 — Supplementary Table S1: Physicochemical Properties of 3DPHA‐PCL vs Bovine Bone. Supplementary Table S2: Summary comparison of 3DPHA‐PCL vs. Bovine Bone in Maxillary Sinus Floor Elevation. [file CRE2-11-e70237-s002.docx]

**Supplementary Table S1**. Physicochemical Properties of 3DPHA-PCL vs Bovine Bone

| **Property** | **3DPHA-PCL** | **Bovine Bone (BB)** | **Measurement Method** |
| --- | --- | --- | --- |
| **Chemical Composition** | HA + PCL (50% wt) | Deproteinized HA | XRD, FTIR |
| **Particle Size** | 2.0 mm spheres | 1.0-2.0 mm granules | Laser diffraction |
| **Total Porosity** | 68.2% ± 4.1% | 72.5% ± 3.8% | Micro-CT |
| **Pore Size Distribution** | Macropores: 50-300 μm | 100-500 μm | SEM |
|  | Micropores: 5-20 μm |  |  |
| **Compressive Strength** | 8.7 MPa ± 1.2 | 3.5 MPa ± 0.9 | Universal testing |
| **Contact Angle** | 82° ± 3° (hydrophilic) | 75° ± 2° (hydrophilic) | Goniometry |

**Supplementary Table S2:** Summary comparison of 3DPHA-PCL *vs*. Bovine Bone in Maxillary Sinus Floor Elevation

| **Category** | **Group BB (Bovine Bone)** | **Group 3DPHA-PCL** |
| --- | --- | --- |
| **Number of Sinuses** | **11** | **11** |
| **Study Design** | Randomized clinical trial across 2 centers | |
| **Imaging Timepoints** | T0 (baseline), T1 (post-op), T2 (6 mo), T3 (1 yr) | |
| **Bone Volume Change (T1–T2)** | −53.9 ± 117.8 mm³ | −40.8 ± 101.2 mm³ |
| **Bone Height Change (T1–T2)** | −0.48 ± 1.01 mm | −0.39 ± 0.44 mm |
| **New Bone Formation (%)** | Histomorphometry: 25.6 ± 7.2%  Micro-CT: 26.3 ± 4.1% | Histomorphometry: 15.7 ± 7.5%  Micro-CT: 16.7 ± 7.5% |
| **Implant Failures** | 0 | 2 |
| **Statistical Significance** | Reference group | - Significantly lower bone formation (*p < 0.05*), - the dimensional stability of the augmented bone was comparable |
| **Conclusion** | Stable volume, superior bone regeneration and implant survival | Comparable volume stability but inferior bone formation and implant success |

**Notes:** T0: Before MSFE, T1: Immediately after MSFE, T2: 6 months (implant placement & biopsy), T3: 1 year follow-up
